# Reframing the science and policy of nicotine, illegal drugs and alcohol – conclusions of the ALICE RAP Project

**DOI:** 10.12688/f1000research.10860.1

**Published:** 2017-03-17

**Authors:** Peter Anderson, Virginia Berridge, Patricia Conrod, Robert Dudley, Matilda Hellman, Dirk Lachenmeier, Anne Lingford-Hughes, David Miller, Jürgen Rehm, Robin Room, Laura Schmidt, Roger Sullivan, Tamyko Ysa, Antoni Gual

**Affiliations:** 1 Institute of Health & Society, Newcastle University, Newcastle upon Tyne, UK; 2 Faculty of Health, Medicine and Life Sciences, Maastricht University, Maastricht, Netherlands; 3 Institute for Mental Health Policy Research, Centre for Addiction and Mental Health (CAMH), Toronto, Ontario, Canada; 4 Centre for History in Public Health, London School of Hygiene and Tropical Medicine, University of London, London, UK; 5 Department of Psychiatry, Université de Montréal, Montreal, Quebec, Canada; 6 Department of Integrative Biology, University of California, Berkeley, California, USA; 7 Center for Research on Addiction, Control and Governance (CEACG), Department of Social Research, University of Helsinki, Helsinki, Finland; 8 School of Social Sciences and Humanities, University of Tampere, Tampere, Finland; 9 Institute for Clinical Psychology and Psychotherapy, TU Dresden, Dresden, Germany; 10 Chemisches und Veterinäruntersuchungsamt (CVUA) Karlsruhe, Karlsruhe, Germany; 11 Centre for Psychiatry, Division of Brain Sciences, Imperial College, Hammersmith Hospital, London, UK; 12 Department of Social & Policy Sciences, University of Bath, Bath, UK; 13 Dalla Lana School of Public Health, University of Toronto, Toronto, Ontario, Canada; 14 Department of Psychiatry, University of Toronto, Toronto, Ontario, Canada; 15 Centre for Social Research on Alcohol and Drugs, Stockholm University, Stockholm, Sweden; 16 Centre for Alcohol Policy Research, La Trobe University, Melbourne, Australia; 17 Institute for Health Policy Studies and Department of Anthropology, History and Social Medicine, School of Medicine, University of California, San Francisco (UCSF), San Francisco, California, USA; 18 Department of Anthropology, California State University, Sacramento, Sacramento, California, USA; 19 Esade-Gov and Department of Strategy, Esade Business School, Ramon Llull University, Barcelona, Spain; 20 Addictions Unit, Department of Psychiatry, Clínic Institute of Neurosciences (ICN), Hospital Clínic, Barcelona, Spain; 21 Institut d'Investigacions Biomèdiques August Pi i Sunyer (IDIBAPS), Barcelona, Spain

**Keywords:** nicotine, illegal drugs, alcohol, evolutionary biology, governance, margins of exposure, well-being, health footprint

## Abstract

In 2013, illegal drug use was responsible for 1.8% of years of life lost in the European Union, alcohol was responsible for 8.2% and tobacco for 18.2%, imposing economic burdens in excess of 2.5% of GDP. No single European country has optimal governance structures for reducing the harm done by nicotine, illegal drugs and alcohol, and existing ones are poorly designed, fragmented, and sometimes cause harm. Reporting the main science and policy conclusions of a transdisciplinary five-year analysis of the place of addictions in Europe, researchers from 67 scientific institutions addressed these problems by reframing an understanding of addictions.  A new paradigm needs to account for evolutionary evidence which suggests that humans are biologically predisposed to seek out drugs, and that, today, individuals face availability of high drug doses, consequently increasing the risk of harm.  New definitions need to acknowledge that the defining element of addictive drugs is ‘heavy use over time’, a concept that could replace the diagnostic artefact captured by the clinical term ‘substance use disorder’, thus opening the door for new substances to be considered such as sugar. Tools of quantitative risk assessment that recognize drugs as toxins could be further deployed to assess regulatory approaches to reducing harm. Re-designed governance of drugs requires embedding policy within a comprehensive societal well-being frame that encompasses a range of domains of well-being, including quality of life, material living conditions and sustainability over time; such a frame adds arguments to the inappropriateness of policies that criminalize individuals for using drugs and that continue to categorize certain drugs as illegal. A health footprint, modelled on the carbon footprint, and using quantitative measures such as years of life lost due to death or disability, could serve as the accountability tool that apportions responsibility for who and what causes drug-related harm.

## Introduction

A consortium of 67 scientific institutions from 24 European countries and beyond, covering over thirty scientific disciplines ranging from anthropology to toxicology, responded to an invitation by the European Commission to study the place of addictions in contemporary European society. The resulting five-year endeavour, the Addictions and Lifestyles in Contemporary Europe - Reframing Addictions Project (ALICE RAP, www.alicerap.eu), went beyond this. It reframed our understanding of addictions and formulated a blueprint for re-designing the governance of addictions. This paper summarizes the project’s conclusions, pointing to new understandings of the science and policy of nicotine, illegal drugs and alcohol, hereafter collectively referred to as ‘drugs’ ^1– 6^. Although this paper does not cover process addictions (e.g., gambling ^3^), much of what is said applies to addictions beyond drugs.

The paper starts by discussing why we need to re-think addictions. It contrasts two powerful pieces of evidence: the harm done by drugs, versus the poorly structured existing governance approaches designed to manage such harm. The paper continues by considering three bases for re-thinking the addiction concept in ways that could lead to improved strategies across different jurisdictions: recognition that there is a biological predisposition for people to seek out and ingest drugs; that heavy use over time becomes a replacement concept and descriptor for the term substance use disorder; and that quantitative risk assessment can be used to standardize harm across different drugs, based on drug potency and exposure. The paper finishes by proposing two approaches that could strengthen addictions governance: embedding governance within a well-being frame, and adopting an accountability system—a health footprint that apportions responsibility for who and what causes drug-related harm.

## Why do we need to re-think addictions?

The need to re-think addictions is exemplified by the extent of harm caused by the drugs themselves, and by the fact that no single country, at least in Europe, has fully overcome poorly managed and fragmented governance structures.

### Harm done by drugs

A standard way to document and describe the interference that drugs have on human biology and functioning is to use years of life lost to premature mortality (YLL) and disability adjusted life years (DALYs). DALYs are a measure of health that sum up YLL and years or life lost due to disability and detriments in functioning (YLD). In 2013, illegal drug use was responsible for 1.8% of YLL in the European Union (EU), alcohol was responsible for 8.2% and tobacco for 18.2% ( Table 1), imposing economic burdens in excess of 2.5% of GDP ^7^.

**Table 1.** Burden of disease caused by drug exposure in the European Union (EU) in 2013. Source: own calculations based on IHME Global burden of diseases, injuries and risk factors study ( http://www.healthdata.org/gbd).

Risk factor	Sex	YLLs in 1,000	YLLs per 100,000	% of all YLLs	DALYs in 1,000	DALYs per 100,000	% of all DALYs
**Illegal drug use**	**Men**	1,069.8	428.5	2.5%	1,749.2	700.7	2.3%
		**Women**	292.7	111.9	0.9%	580.5	222.0	0.8%
		**Total**	1,362.5	266.6	1.8%	2,329.7	455.8	1.6%
**Alcohol use**	**Men**	4,558.7	1,826.1	10.4%	5,981.4	2,396.0	7.9%
		**Women**	1,584.0	605.8	5.1%	2,019.8	772.5	2.9%
		**Total**	6,142.8	1,201.9	8.2%	8,001.2	1,565.5	5.5%
**Tobacco use**	**Men**	10,036.4	4,020.3	23.0%	11,280.0	4,518.5	14.9%
		**Women**	3,552.2	1,358.6	11.5%	4,405.0	1,684.7	6.4%
	****	**Total**	13,588.6	2,658.6	18.2%	15,685.0	3,068.8	10.9%

YLL: Years of life lost due to premature mortality DALYs: Disability adjusted life years Source data available in Dataset 1 ^102^.

The data in Table 1 represents harm to the drug user. However, drug use also harms the health of others. For instance, operating machinery under the impact of illegal drugs can cause injury to others ^8, 9^. Although decreasing globally, second-hand smoking was estimated to kill more than 330 thousand people worldwide in 2013, and caused about 7% of the burden of disease in DALYs attributable to tobacco smoking ^10^. The extent of harm to others caused by alcohol consumption is estimated to be proportionally even larger, mainly due to traffic accidents, violence, including homicide, and foetal alcohol spectrum disorders ^11^.

Source data underlying the results presented in Table 1 Click here for additional data file. Copyright: © 2017 Anderson P et al. 2017 Data associated with the article are available under the terms of the Creative Commons Zero "No rights reserved" data waiver (CC0 1.0 Public domain dedication).

### Fragmented governance structures

Governance is defined as the processes and structures of public policy decision making and management that engage people across the boundaries of public agencies, levels of government, and public, private and civic spheres to carry out a public purpose that cannot be accomplished by any one sector alone ^12^. The involvement of multiple stakeholders in governance is not without risk. The exclusive use of top-down bureaucratic approaches cannot maximize societal benefits when dealing with ‘wicked problems’ that are highly resistant to resolution ^13^ (for definition of wicked problems, see ‘The New Governance of Addictive Substances and Behaviours by Anderson *et al* ^6^). An analysis of 28 European countries found that no single country had a comprehensive policy for all drugs (including nicotine, illegal drugs and alcohol) within a broad societal well-being approach. For more detail, see ‘Governance of Addictions: European Public Policies’, by Albareda A *et al* ^1^.

There are at least three reasons for ineffective and poorly integrated governance. Firstly, the same harm done by drugs is defined and understood in different ways in different countries and state systems ^14– 16^. Seen from a trans-national comparative perspective, there is a lack of a common understanding of appropriate policies, and responses are often constrained by approaches that are tied to assumptions that are not evidence-based ^4^. Ways of thinking about the harm done by drugs vary enormously, with considerable heterogeneity between different drugs, and between international, national and local levels of governance. For detail, see ‘Concepts of Addictive Substances and Behaviours across Time and Place, by Hellman *et al* ^4^.

Secondly, a multitude of commercial, political and public stakeholders are active in addictions governance on national and international levels. In any given society, stakeholders that have power, means and influence are likely to achieve an advantageous influential position. The concepts of addiction are also shaped by popular constructs promulgated by the mass media and customs in the general population. Stakeholder positions and perceptions of drug problems also vary over time and by area ^4^, implying that sustainable approaches must be interwoven into societal and governance structures.

Thirdly, corporate power ^17^, through multiple channels of influence, can hinder evidence-based policy decisions ^5^. Corporate strategies often include attempts to influence civil society, science and the media, as part of a wider aim to manage and, if possible, capture institutions that set policy. Transparency is insufficient given that the multiplicity of channels with corporate power is poorly acknowledged and understood by policy makers. Therefore, the rules in place to ensure level playing fields for discussions and equitable decision-making across all factors are inadequate ^6^.

## Reframing addictions

The consensus reached under ALICE RAP was that there are at least three ways to reframe addictions that could lead to improved strategies across different jurisdictions. These include:

1) Recognition that humans have a biological predisposition for seeking out and ingesting drugs;

2) Recognition that ‘heavy use over time’ should replace the concept and term ‘substance use disorder’;

3) Recognition that a quantitative risk assessment accounting for drug potency and drug exposure, can standardize measures of harm across different drugs.

### Evolutionary evidence for biological predisposition

The idea that human exposure to drugs did not occur until late in human evolution—thus leaving our species inexperienced—is often posited as one of the reasons that these substances cause so much harm ^18^. However, multidisciplinary scientific evidence suggests otherwise. Many substances consumed today are not evolutionary novelties ^18, 19^. In the story of terrestrial life over the last 400 million years or so, one ongoing theme has been the “battle” between plants and the animals that eat them. Of the many defence mechanisms in existence, plants produce numerous chemicals, including tetrahydrocannabinol, cocaine, nicotine, and opiates, all of which are potent neurotoxins that deter consumption of plant tissue by animals ^18^. From an evolutionary perspective, we thus find natural selection for compounds that discourage consumption of the plant via punishment of potential consumers. By contrast, there has been no natural selection for expression of psychoactive compounds which encourage consumption (i.e., via consumer reward), which has also been predicted by neurobiological and behavioural psychology theories of reward and reinforcement for contemporary drugs ^20^.

Counterbalancing the development of plant neurotoxins, plant-eating animals have evolved to counter-exploit plants’ production of drugs, for instance by exploiting the anti-parasitic properties of some of them ^18^. Many species of invertebrates and vertebrates engage in pharmacophagy, the deliberate consumption and sequestration of plant toxins, to dissuade parasites and predators. In a human context, present day examples of pharmacophagy may be seen with Congo basin hunter gatherers, amongst whom the quantity of cannabis ^21^ and nicotine ^22^ consumed is titrated against intestinal worm burden - the higher the intake, the lower the worm burden. In individuals treated with the anti-worm drug abendazole, the number of nicotine-containing cigarettes smoked is reduced ^22^.

Although parasite-host co-evolution is recognized as a potent selective force in nature, other, subtler evolutionary dynamics may affect human and animal interactions with plant-based drugs, including that they may buffer against nutritional and energetic constraints on signalling in the central nervous system ^23^. Ethnographic research reveals that many indigenous groups classify “drugs” as food, rather than psychoactive entities, and that they are perceived as having food-like effects, most notably for increasing tolerance for fatigue, hunger and thermal stress in nutritionally-constrained environments ^23^. The causes of these perceived effects have not been a research question, but there are clues that the “food” classification may be literal rather than allegorical. Common plant toxins not only mimic mammalian neurotransmitters, they are also synthesized from the same nutritionally-constrained amino acid precursors, such as tyrosine and tryptophan. In harsh environments, toxic plants could function as a “famine food” providing essential dietary building blocks, or, may function as a direct substitute for nutritionally-constrained endogenous neurotransmitters. There is some evidence to support this hypothesis in animal research; for example, wood rats in cold environments reduce thermoregulatory costs by modulating body temperature with plant toxins consumed from the juniper plant ^24^.

In the case of ethanol, its presence within ripe fruit suggests low-level but chronic dietary exposure for all fruit-eating animals, with volatilized alcohols potentially serving in olfactory localization of nutritional resources (i.e., animals can use the smell of alcohol to locate food over long distances) ^19^. Molecular evolutionary studies indicate that an oral digestive enzyme capable of rapidly metabolizing ethanol was modified in human ancestors near the time that they began extensively using the forest floor, about 10 million years ago ^25^; humans have retained the fast-acting enzyme to this day. By contrast, the same alcohol dehydrogenase in our more ancient and mostly tree-dwelling ancestors did not oxidize ethanol as efficiently. This evolutionary switch suggests that exposure to dietary sources of ethanol became more common as hominids adapted to bipedal life on the ground. Ripe fruits accumulating on the forest floor could provide substantially more ethanol cues and result in greater caloric gain relative to fruits ripening within the forest canopy, and our contemporary patterns of alcohol consumption and excessive use may accordingly reflect millions of years of natural dietary exposure ^19^.

This evolutionary evidence does not imply that humans also evolved to specifically consume nicotine, for example, or that nicotine use is beneficial in the modern world. What is novel in the modern world is the high degree of availability, and high concentration of psychoactive agents and routes of consumption that promote intoxication. What is different with alcohol in the modern world is novel availability through fermentative technology, enabling humans to consume it as a beverage, devoid of food bulk, with higher ethanol content, and artificially higher salience than that which characterizes fruit fermenting in the wild. The evolutionary evidence has two implications: firstly, policies that prohibit the use of drugs are likely to fail because people have a biological predisposition to seek out chemicals with varying nutritional and pharmacological properties; and secondly, in present-day society, drug delivery systems have been developed that are beyond what is reflected in the natural environment, particularly with respect to levels of potency, availability and taste, which could be argued as being the more central drivers of harm. Potency is largely determined by producer organisations operating in markets, which, from the perspective of overall societal well-being, are inadequately managed ^26^. Better regulation of potency can become a major opportunity for additional policy interventions - for example with alcohol, see ‘Evidence of reducing ethanol content in beverages to reduce harmful use of alcohol’ by Rehm *et al*. ^27^.

### Heavy use over time

To better understand the interference of drugs in human biology and functioning, the consensus reached in ALICE RAP was that the concept and term ‘heavy use over time’ should be proposed as the replacement for ‘substance use disorder’. In medical settings and indeed often in academic and lay settings, heavy users of drugs are commonly dichotomized into those with a ‘substance use disorder’ or not. ‘Substance use disorder’ is a clinical construct that is often used as a shorthand to identify individuals who might benefit from advice or treatment. But as a condition in itself, it is a medical artefact which occurs in all grades of severity, with no natural distinction between ‘health’ and ‘disease’ ^28, 29^.

This is illustrated with alcohol. The associated chronic organ damage (e.g., liver cirrhosis, cancers) exponentially increases in risk as alcohol consumption accumulates over time ^30, 31^. Unmanaged heavy drinking is associated with subsequent heavy drinking, often culminating in brain damage ^32^, itself a consequence of heavy drinking ^33, 34^ but also a driver of future behaviour.

Alcohol consumption itself is close to log-normally distributed in drinking populations, skewed towards heavy drinking ^35^. There is no natural cut-off point above which "alcohol use disorder" definitively exists and below which it does not. “Alcohol use disorder” is clinically defined as a score on a checklist of symptoms, and there is a smooth line exponential relationship between levels of alcohol consumption and the score on the checklist ^29, 36^. Heavy drinking is a cause of the items on the checklist, including compulsion to drink more, which can also be a consequence of brain damage, itself caused by heavy drinking. Thus, “alcohol use disorder” is a diagnostic artefact. No more is needed to consider what is called “alcohol use disorder” other than heavy use over time ^28, 29^.

For alcohol (and other drugs as well), this approach does not imply that heavy use over time is the only cause of harm. There are other factors involved that that drive heavy alcohol use and harm ^3^ that are independent of, or in interaction with, molecular and cellular levels (e.g., alcohol dehydrogenase polymorphisms ^37^), individual levels (e.g., income ^38^ and personality ^39^) and environmental levels (e.g., stigma)

There is an ongoing discussion as to whether or not sugar is an ‘addictive’ substance that should be captured in the same category as drugs ^26^. Framing the problem as one of heavy use over time provides insight into this debate. As with alcohol and high blood pressure ^40^, chronic disease risk associated with plasma glucose levels has a continuous exponential relationship with sugar consumption ^41^. The distribution of blood glucose levels is close to log-normally distributed in populations and skewed towards high consumption levels ^42^. There is no natural cut-off point above which diabetes (or any other disease manifestation) linked to sugar definitively exists and below which it does not. Similar to the alcohol model where heavy use of alcohol over time leads to further heavy use of alcohol from the resulting brain damage, heavy use of sugar over time damages hippocampal function ^43^, which leads to further heavy use of sugar over time ^44^. Thus, in the ‘heavy use over time’ frame, sugar can be placed in the same category as alcohol and other drugs, and managed with similar governance approaches that promote public health.

### Quantitative risk assessment

A core way to document the interference of drugs in human biology and functioning is to use quantitative risk assessment (QRA). QRA is a method applied in regulatory toxicology, for example, to evaluate water contaminants, and before safety approvals for food additives or pesticides. QRA has not been widely applied to drugs. Previous approaches for ranking harm have mostly been based on expert judgements ^45, 46^ which have been criticized as being arbitrary and biased ^47^.

The advantage of QRA is that it provides a formal scientific method to rank the harm-potential of drugs, making optimum use of available data ^48^. There are several approaches for QRA available, with Margin of Exposure (MOE) suggested by WHO ^49^ as being most suitable for prioritizing risk management. In the alcohol field, MOE has been applied to evaluate the liver cirrhosis risk of ethanol, which is the single most important chronic disease condition attributable to alcohol globally ^50^. MOE results have replicated those behind existing guidelines for low-risk drinking ^51^. In a detailed study of the components in alcoholic beverages, ethanol was confirmed as the compound with highest risk ^52^. In a detailed comparison between ethanol and non-metabolically produced acetaldehyde contained in beverages, it was also judged that the risk of ethanol comprises more than 99% of the total risk ^53^. It can be concluded that the risk of alcoholic beverages can be evaluated by looking at the effects of ethanol alone. The situation is less clear for tobacco, for which some industry MOE studies find toxicants other than nicotine ^54, 55^. An MOE analysis of electronic cigarette liquids indicated that nicotine is the compound posing the highest risk ^56^.

**Figure 1.**
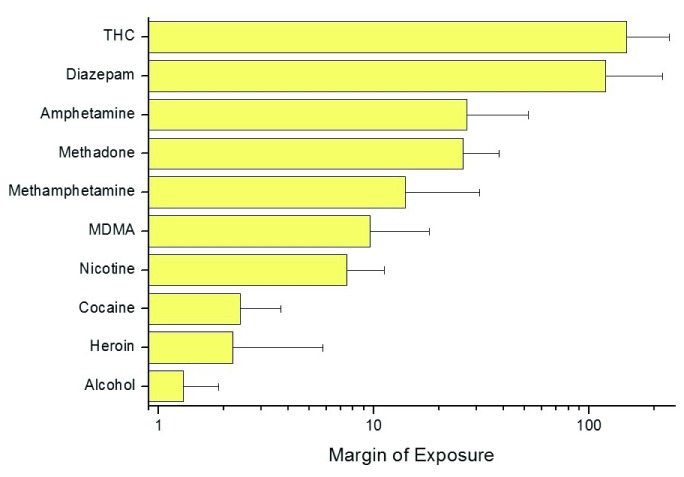
Margin of exposure for daily drug use estimated using probabilistic analysis. Source: Lachenmeier & Rehm (2015) ^47^.

MOEs are calculated as the ratio of a toxic dose of the drug (usually the benchmark dose BMDL10, the lowest dose which is 95% certain to cause no more than a 10% negative outcome incidence) with the dose consumed either individually or on a population scale ^47^. The higher the MOE, the lower the level of risk, with low risk not implying safety. An MOE of 100 means that the drug is being consumed at one hundredth of the benchmark dose; an MOE of 1 means that the drug is being consumed at this toxic dose. The MOE for drugs can be calculated taking into account a range of hazard outcomes in health and other well-being domains, as long as suitable dose-response data are available (which is not the case for most drugs and many well-being indicators). Therefore, analyses to date are primarily restricted to lethal outcomes based on animal studies. Results for European adults are summarized in Figure 1. The low MOE for alcohol (and thus high risk) is due to the high levels of consumption by European adults. The MOE results are consistent with the consensus of expert rankings in which cannabis is ranked with lower risk and alcohol with higher risk than current policies assume ^45, 46^. The MOE is inherent to the drug itself; it does not account for the harms that arise from drug delivery systems, for example, smoked tobacco, or from secondary effects such as unclean syringes used for heroin intake.

Of course, MOE, as presented here, focuses on the physical body of the adult user as the locus of harm. It does not take into account the sex and age of the user, or harm to individuals other than the user or at collective levels, which are a primary source of social differentiation between drugs. It also focuses on mortality, rather than intoxication in the moment. Differences between the intoxicating power of substances in the moment, and in the behavioural consequences of taking them, are primary reasons why, for example, societies have treated alcohol differently to tobacco. Nevertheless, we believe that MOE should be applied at the current stage even when the underlying toxicological data are incomplete, to provide a better alignment of prioritization of policy to the drugs associated with higher risks, which in this case are nicotine, cocaine, heroin and alcohol.

## Towards better governance

We have described three harmonizing approaches to reframe our understanding of addictions: biological predisposition to seek out psychoactive substances; heavy use over time as a fruitful characterisation; and quantitative risk assessment. Here, we propose two underlying pillars for a re-design of the governance of drug controls: embedding drugs governance within a comprehensive model of societal well-being; and creating a health footprint which, modelled on the carbon footprint, promotes accountability by identifying who causes what harm to whom from drugs.

### Societal well-being

**Figure 2.**
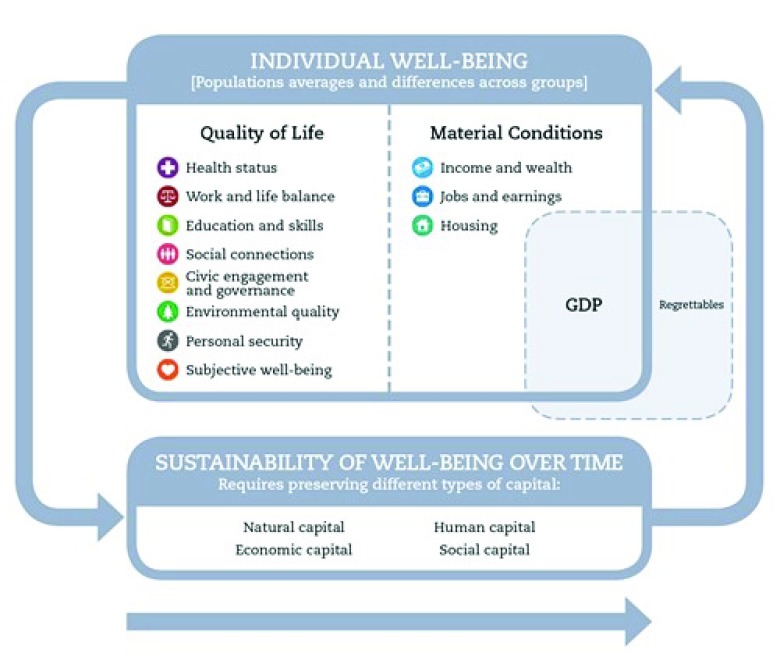
Well-being framework, reproduced with permission from the OECD Better Life Index initiative. Source: OECD. (2011), How's Life?: Measuring Well-being, OECD Publishing, Paris. DOI: http://dx.doi.org/10.1787/9789264121164-en ^57^.

We propose that societal well-being should be our overarching frame for a more integrated governance and monitoring of drug control policies. Societal well-being, as captured by OECD ^57^, includes quality of life (health, education and skills, social connections, civic engagement, and personal security), material conditions (income, employment and housing) and sustainability over time (see Figure 2). Gross domestic product (GDP) is included as a separate domain, recognizing that, while economic well-being is an important component of societal well-being, GDP has significant limitations. GDP excludes, for example, non-market household activity such as parenting, and activities such as conservation of natural resources. GDP also includes activities which do not contribute to well-being, such as pollution and crime, termed regrettables that are depicted within GDP but outside well-being. The use of and harm done by drugs are affected by and affect all well-being dimensions ^58^.

Well-being analyses have found that, whilst some illegal drug policies may reduce health harms, they often come with adverse side effects including criminalization, social stigma and social exclusion, all of which exacerbate health harms ^59^. Humans are hard-wired to be social animals ^60^, with social networks strongly influencing tobacco use ^61^ and alcohol intake ^62^. Punitive drug policies bring about the opposite: social exclusion due to stigma and social isolation ^63– 65^. Engagement with illegal drugs conveys especially strong social meanings and can lead to stigma of marginalized heavy users, as opposed to the supposedly more responsible mainstream users ^66^. This can lead to punitive societal responses. Meanwhile, exclusion from the mainstream may allow harms to continue unchecked. If a user is caught using drugs in a country with “zero tolerance” to illegal drugs, the ensuing criminal sanctions will impede civic engagement and any improvements in quality of life and material living conditions. For more detail, see ‘Well-being as a frame for understanding addictive substances’ by Stoll & Anderson ^58^. Changes in life expectancy in Mexico illustrate the negative consequences of criminalization ^67^. After six decades of gains in life expectancy in Mexico, the trend stagnated after 2000 for both men and women, and for men was reversed after 2005 ^68^. This was largely due to an unprecedented rise in homicide rates, mostly as a result of drug policies promoting ‘gang wars’ and conflicts between gangs, the police and army ^69^.

A well-being frame calls for whole-of-society approaches that progressively legalize illegal drugs to reduce violence and personal insecurity, while focusing on substances as drivers of harm ^6, 70^. It balances the complex factors impacting drug use and related harm through the continuous monitoring of policy effects in a proactive way, with regulations embedded in international coordination. It calls for whole-of-society approaches that avoid criminalization where possible and where costs of addressing the problem are equally distributed across society. Governance strategies manage nicotine, illegal drugs and alcohol as a whole to avoid overlaps, contradictions, gaps and inequalities ^1^. The concern should be focused on harms, both to the user and to others, including family and friends, communities and society as a whole. The structures to support the strategies should be coordinated and multi-sectoral, involving high-level coordination of health, social welfare, and justice agencies in the context of international treaties, and, importantly, equitable across the lifespan, between genders and cultural groups. To increase the pace of policy change, regional and local public policies can create *policy communities* and networks within a common international framework.

Managing ‘wicked problems’ requires clear rules of private sector engagement in policy making, particularly when private interests go against societal well-being ^6^. An evolved governance system must include measures to avoid industry co-optation, through transparency, checks and balances. Private sector stakeholders should operate within established rules.

### Accountability

The ongoing monitoring of outcomes within a well-being framework would promote accountability. Modelled on the carbon footprint, we propose a health footprint as the accountability tool. Footprints were developed in the ecological field as a measure of human demand on ecosystems ^71^, including water footprints ^72^ and carbon footprints that apportion greenhouse gas emissions to certain activities, products and populations ^73^. The central reason for estimating a carbon footprint is to help reduce the risk of climate change through enabling targeted and effective reductions in greenhouse gas emissions ^74^.

**Figure 3.**
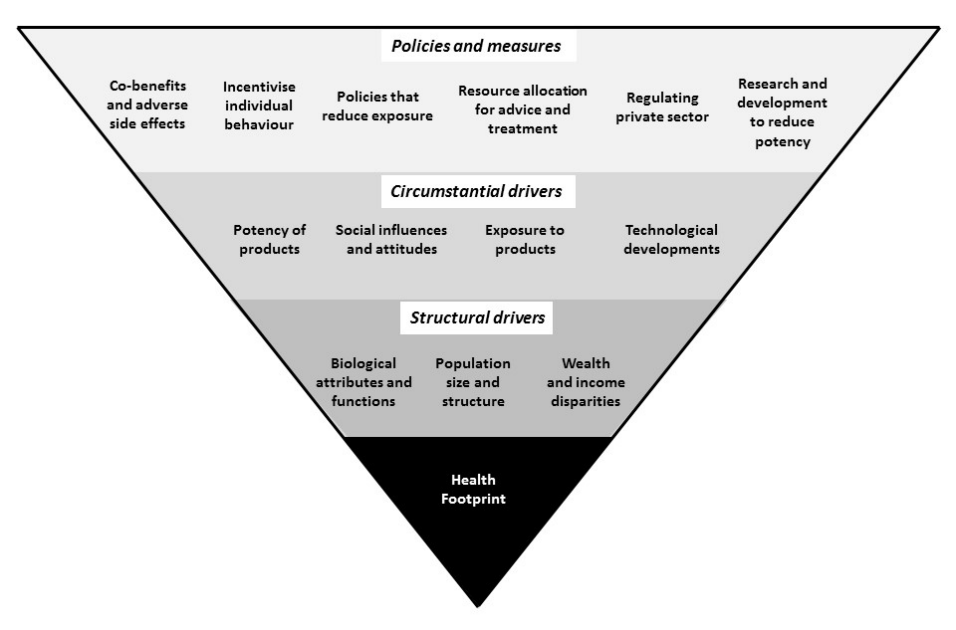
Drivers of harm done by drugs and addictive behaviour.

The health footprint can be considered a measure of the total amount of risk factor attributable disability adjusted life years (DALYs) ^75^ of a defined population, sector or action within a spatial (e.g., jurisdiction) or temporal boundary (e.g. one year). It can be calculated using standard risk factor-related YLL and DALY methodologies of the Global Burden of Disease Study ^10^ and of the World Health Organization ^75^. Health footprints are a starting point. To be accountable, we ultimately need to understand what drives the health footprint ( Figure 3).

### Structural drivers

Above the health footprint of Figure 3 are the structural drivers of harm that directly influence the size of the health footprint. Biological attributes and functions include, for example, the biological pre-disposition to seek out and use drugs. Genetic variants, for example, could be those that affect the function of alcohol dehydrogenase, influencing consumption levels and harm ^8, 76^. Changes in global population size and structure can increase absolute numbers of drug-related DALYs, even though rates per person can decrease over the same time ^7^. As sociodemographic status improves in lower income countries, so do drug-related DALYs ^10^; yet, for the same amount of drug use, people with lower incomes suffer more drug-related DALYs than people with higher incomes ^77^.

### Circumstantial drivers

Above the structural drivers are the circumstantial drivers, those that can change. Related to drug potency and exposure, an MOE target for all drugs no greater than 10 has been argued ^6^. Policies could achieve such a result by either reducing drug exposure or by reducing the potency of the drug. Technological developments have led to electronic nicotine delivery systems (widely known as e-cigarettes) as widespread alternatives to smoked tobacco, with current best estimates showing e-cigarettes to be considerably less harmful to health than smoked cigarettes ^78– 80^. It may be that once e-cigarettes are heavily produced and marketed by the tobacco industry, that society will see cigarette-like levels of sustained heavy use of nicotine. However, e-cigarette’s harm quotient should stay low, provided they are properly regulated in terms of their components, including nicotine. Social influences and attitudes drive harm through stigma, social exclusion and social marginalization; these are often side-effects of drug policies, which can bring more harm than drug use itself ^81, 82^.

### Policies and measures

Policies that reduce exposure to drugs are essentially those that limit availability by increasing the price and reducing physical availability ^59, 83, 84^. The absence of such evidence-based policies is an important driver of harm. Limits to availability bring a range of co-benefits to educational achievement and productivity, for example, but they can also bring adverse effects – for example, the well-documented violence, corruption and loss of public income associated with some existing ‘illegal’ drug policies ^58, 85^. Individual choices and behaviour that drive harm from drug use are determined by the environment in which those choices and behaviours operate ^86^. Banning commercial communications, increasing price and reducing availability are all incentives that impact individual behaviour. Research and development can be promoted to reduce the potency of existing drugs ^87^ and their drug delivery packages ^27, 56, 78^.

Unfortunately, there remain enormous gaps between the supply and demand of evidence-based prevention, advice and treatment programmes ^88– 92^. Called for by United Nations Sustainable Development Goal 3.5 ^93^, their supply can bring many co-benefits to society, including reduced social costs and increased productivity ^94^. The harm driven by the gaps is due in large part to insufficient resources and insufficient implementation of effective evidence-based prevention and treatment programmes ^95^. Currently these programmes represents less than 1% of all costs incurred to society by drugs ^96^. Similar to medicines agencies (such as the European Medicines Agency) that assess and approve drugs, prevention agencies could be created ^95^. Compounding the gap between supply and demand is the fact that often, considerable marginalization and stigmatization happens in the path to treatment, and this is then further exacerbated by the treatment itself ^82^. The use of pharmacotherapy as an adjunct may be further limited due to ideological stances, poorly implemented guidelines, lack of appropriate medication, and even a perceived lack of effect, if the drug is available ^97^.

The private sector is a core driver of harm, through commercial communications which include all actions undertaken by producers of drugs to persuade consumers to buy and consume more ^98^. There are international models encouraging better control of commercial communications in the public health interest, the most notable being the Framework Convention on Tobacco Control ^83^. In addition to commercial communications, the private sector drives harm through shaping drug policies, leading to more drug-related deaths ^5^. Governance structures thus need to have the capability and expertise to supervise industry movements that shape drug-related legislation and regulations, including regulating and restricting political lobbying. One of the difficulties here is that politically driven change in difficult areas, such as drug policies, is highly dependent on collective decisions ^99^ and influenced by what has been termed specular interaction ^100^, in which a politician’s actions may be less determined by their own conviction, and more by their evaluation of beliefs of their rivals and friends.

The health footprint is the accountability system for who and what causes drug-related harm. Jurisdictional entities can be ranked according to their overall health footprint, in order to identify the countries that contribute most to drug attributable ill-health and premature death, and where the most health gain could be achieved at country level. Jurisdictional footprints could include ‘policy attributable health footprints’ which estimate the health footprint between current policy and ideal health policy. This would address the question: ‘what would be the improvement in the health footprint compared to present policies, were the country to implement strengthened or new policies?’ Conversely, the health footprint can provide accountability for when such evidence-based policy is not implemented correctly.

A range of sectors are involved in nicotine and alcohol related risk factors. These include producer and retail organizations such as large supermarket chains, and service provider companies such as advertising and marketing industries. There is considerable overlap between sectors, and estimates will need to determine appropriate boundaries for health footprint calculations. Companies could report their health footprints and choose to commit to reducing them by a specified amount over a five to ten-year time frame. Direct examples of producer action could include switching from higher to lower alcohol concentration products ^27^, and switching from smoked tobacco cigarettes to e-cigarettes ^80^.

## Conclusions

The points stated above underscore the need to redesign the governance of drugs; in Europe, and globally. Margins of exposure estimates for four drugs (nicotine, cocaine, heroin and alcohol) are exceedingly high and thus call for determined action. Drugs are responsible for a high proportion of years of life lost in the European Union; tobacco accounted for 18.2% of life years lost, illegal drugs for 1.8%, and alcohol for 8.2% in 2013. There are many side effects of existing policies, such as stigma, social exclusion, lack of personal security, civil unrest and homicide ^58^.

Under the auspices of ALICE RAP, a large, multidisciplinary team of addiction scientists put forward a range of arguments for moving progressively towards regulated legalization of certain illegal drugs, proposing a well-being frame that calls for whole-of-society approaches and continuously monitors and accounts for adverse side effects of drug policy. Humans have a biological pre-disposition to seek out a range of drugs, so prohibitionist policies are likely to run into difficulty - and they have. Legalization does not imply that drug governance is left to market forces alone - the experience of nicotine and alcohol demonstrates that this is not possible. Instead, drug governance requires comprehensive regulation, with adequate and transparent rules of the game for stakeholder involvement, and appropriate international regulatory frameworks. With a health footprint, it can be documented who causes what harm from nicotine, illegal drugs and alcohol in the public and private sectors. Public bodies and private companies should be required to publish their health footprints on an annual basis, and indicate their plans for reducing the health footprint.

The consensus that ALICE RAP reached will not come without push-back. Without input from evolutionary theory, neurobiology will continue to maintain that human drug use is initiated and sustained by reward and reinforcement at both biological and behavioural levels, compounded by mistaken views that the human encounter with drugs is a relatively new evolutionary experience, and human vulnerability to drugs in moral, behavioural, and biological dimensions. Disease classification systems are based not only on measurement, but on qualification, and thus payment, for treatment. The concept heavy use over time does not prevent the use of qualification definitions for treatments. Threshold consumption levels determining treatment can be defined as levels above which advice and treatment have been shown to reduce the development or progression of end-organ damage. Extending margin of exposure analyses for a range of outcomes beyond mortality will overcome concern of one metric for drug policy - its strength is that it allows standard comparison across drugs and indicates options for changing both dose and exposure.

Whilst measuring societal well-being as a whole has gained support, the implications for drug policy that favour regulated legalization will meet resistance from those who favour prohibition, particularly as prohibition is based more on a moral than an evidence-based standpoint, as has been the case with alcohol ^101^. The footprint implies responsibility, which is often difficult for both public and private sectors to accept, in particular for producer companies whose vested interests might be challenged.

What we propose in this paper are large adjustments to our understanding of addictions and to what needs to be done to effectively reduce the widespread harms done by drugs. We hope that what we have written might start a process for better drug policy for the good of the public.

## Data availability

The data referenced by this article are under copyright with the following copyright statement: Copyright: © 2017 Anderson P et al.

Data associated with the article are available under the terms of the Creative Commons Zero "No rights reserved" data waiver (CC0 1.0 Public domain dedication). 

**Dataset 1: Source data underlying the results presented in Table 1.** The data was based on the IHME Global burden of diseases, injuries and risk factors study ( http://www.healthdata.org/gbd).

DOI, 10.5256/f1000research.10860.d154573 ^102^
