# Supplementary material for: Reframing the science and policy of nicotine, illegal drugs and alcohol – conclusions of the ALICE RAP Project
Source: F1000Res. 2017 Mar 17;6:289. [Version 1] doi: 10.12688/f1000research.10860.1 (PMC5381624; doi:10.12688/f1000research.10860.1)
Supplement: Peer review history

# Peer review: Referee response for version 1

**DOI:** 10.5256/f1000research.11710.r21515

**Published:** 2017-04-03

**Authors:** Freya Vander Laenen, Nick Verhaeghe

**Affiliations:** 1 Institute for International Research on Criminal Policy (IRCP), Ghent University, Ghent, Belgium; 2 Department of Public Health, Ghent University, Ghent, Belgium

The opinion article on reframing and rethinking the science and policy of nicotine, illegal drugs and alcohol is an interesting article that challenges some of the current ‘frames’ to analyze addictive substances and the governance of these substances. The surplus value of the article lies in the insights provided from a truly interdisciplinary approach, including disciplines beyond the ‘usual suspects’ of health economics, criminology and psychology. It will stimulate discussing on governance approaches that promote public health.

 Overall, this is a well-written article addressing the important topic of reframing the science and policy of nicotine, illegal drugs and alcohol. The main messages of the paper are clearly described and sufficiently accentuated.

 There is one important opinion that is included in the article that should be substantiated more extensively though. On p. 7 (final paragraph) and again in the overall conclusion, the authors plea to ‘legalize’ (p. 7) illegal drugs and they plea for approaches ‘that avoid criminalization’; in the conclusion, gain they plea for a ‘regulated legalization’ (p. 9), and for a ‘legalization (p. 10). First, the concepts used are not synonymous, as they are different legal concepts with a different meaning/ with different implications. Avoiding criminalization is not the same thing as legalization (avoiding criminalization does not require legalization, but can be reached through depenalization or by making use of the expediency principle on the prosecution level to settle drug offences); neither is ‘regulated legalization’ and ‘legalization’ (neither alcohol nor tobacco are legalized for that matter). I would advise the authors to reconsider the terms used and to extend on the implications of the option they suggest. Second, and linked to this, the transition from the paragraph on the consequences of criminalization (in Mexico) and the plea for legalization on p. 7 is too abrupt.  

 Next, there are some minor questions that arise at some paragraphs in the article we would like the authors to elaborate on.

 On page 3, section ‘Harm done by drugs’, the authors refer to the use of DALYs as a standard way to quantify the harm caused by drugs. The authors propose to use DALYs as a measure for the health footprint. I agree with this since the use of DALYs enables to make comparisons associated with the burden of drugs across substances and/or countries possible. It is well known that the (mis)use of drugs result in an increased risk of a number of conditions (somatic diseases, mental disorders, injuries). Relative risks (together with prevalence data) can serve as input to estimate substance-attributable fractions (SAFs) which can be used to quantify the economic burden of  drug (mis)use. So, please elaborate a bit on this in the section ‘Harm done by drugs’.

 On p. 5, the authors state that the heavy use over time of sugar can be placed in the same category as alcohol and other drugs. Do the authors imply that heavy use over time of sugar should be governed to the same extent as, e.g., the heavy use of heroin (and that heavy use should be the main element in policy decisions)? Or do the authors plea for a differentiated  substance policy between different types of substances? Does this mean that the focus should be on rewarding healthy lifestyle behavior? In addition, policy initiatives to reduce the use of sugar should be integrated together with other lifestyle-related interventions such as the promotion of more physical activity, healthy eating (not restricted to only reducing the use of sugar). Please, add some comments on this.

 On p. 6, the authors state that suitable dose-response data have to be available and they continue to state ‘which is not the case for most drugs and many well-being indicators’. Could the authors add what the main reasons are why these data are missing and how this lack of data could be overcome?

 On p. 6, the authors only briefly touch upon the intoxicating power of substances in the moment and upon the behavioral consequences of taking them. One might argue that taking one of these elements is suitable for prioritizing risk management as well? Could the authors thus more clearly argue why, they suggest to use MOE and not, e.g. intoxicating power?

 On page 9 section ‘Policies and measures’, ‘Banning commercial communications’, ‘increasing price’, and ‘reducing availability’ are incentives that impact individual behavior. Generally, these incentives could be considered as more or less ‘restrictive incentives’ that impact individual behavior. On the other hand, incentives can also be considered as ‘rewards’. What do the authors think about e.g. the use of financial incentives to reward ‘healthy behavior’? How could/should these be incorporated into an integrated governance approach?

 Some of the concepts in figure 3 are not explicitly discussed in the article, or at least not in a logical/sequential order (e.g. regulating private sector and research and development are, resource allocation and incentivizing individual behavioral are not). Could the authors briefly discuss each of the elements in this interesting figure? Could the authors please elaborate a bit more on the advantages (added value) and limitations of the conceptual model?

We have read this submission. We believe that we have an appropriate level of expertise to confirm that it is of an acceptable scientific standard.

# Peer review: Referee response for version 1

**DOI:** 10.5256/f1000research.11710.r21185

**Published:** 2017-04-03

**Authors:** Richard L. Bell

**Affiliations:** 1 Department of Psychiatry, Indiana University School of Medicine, Indianapolis, IN, USA

The present manuscript by Anderson *et al*. "Reframing the science and policy of nicotine, illegal drugs and alcohol...." is a well-written succinct, compilation of the findings and suggestions obtained from the Addictions and Lifestyle in Contemporary Europe--Reframing Addictions Project (ALICE-RAP).  The authors, and colleagues of associated publications, astutely highlight the need for a systematic lexicon for addiction science and policy.  This lexicon is needed not only internationally but also intranationally at all levels of the private and public sector.  Thus, the need to destigmatize addiction and recognize that it is a natural phenomenon requiring treatment, and not criminalization, in some individuals.  This will require a "reframing of addiction" in order to facilitate the treatment of addiction.  While progress has been made in recognizing that addiction is a medical condition, that progress has not been matched by efforts to destigmatize addiction.  As long as addiction is not recognized as a natural phenomenon that isn't isolated to one substance, but includes multiple licit and illicit substances (and possibly behaviors/process addictions), the public and policy makers will continue to have a mind set that addiction represents a "wicked problem".  Thus, addiction will continue to be criminalized with most funds targeting addiction policy being slated towards the criminal justice system rather than social and clinical medicine to treat the phenomenon.  The authors put forth a hypothesis that the lack of consistent addiction policy not only internationally but also intranationally is the absence of a standardized measure of social, medical, economic, and civic damage resulting from addiction to different licit and illicit substances.  Disability Adjusted Life Years (DALYs) is a recommended way to address the health impact disparity observed across different classes of substance with abuse potential.  DALYs can be used to determine Margin of Exposure (MOE) as a Quantitative Risk Assessment (QRA), which can be standardized across "substances of abuse".  When this is done, as seen in Figure 1, it is clear that the licit substances ethanol and nicotine have a significantly greater deleterious "health footprint" compared to most illicit drugs.  Yet, global addiction policy is not consistent in recognizing, nor addressing, this disparity.  Finally the authors recognize and remind the reader that addiction policy is influenced by social, political and market place suppositions that are not evidence-based.  Moreover, with the nontransparency of lobbying activity by multiple, and diverse, stakeholders on policy makers will resist a "reframing of addiction" in order for there to be consistent, equitable and humane policy both internationally and intranationally. 

 As far as particular manuscript content goes, the authors discuss the relatively novel concept that plant neurotoxins, which many drugs of abuse mimic or contain, are evolutionarily conserved in the plant kingdom.  Thus, these toxins dissuade animals from ingesting the plant.  Contrarily, plant chemicals that promote ingestion, in and of themselves, through reinforcing and/or rewarding effects are evolutionarily "weeded out".  This point is receiving greater support through the recognition that immune signaling, centrally and peripherally, plays an important role in the neurobiology of addiction.

I have read this submission. I believe that I have an appropriate level of expertise to confirm that it is of an acceptable scientific standard.
